# Chronic use of low dose simvastatin does not alter the IVCT in normal rats

**DOI:** 10.1186/1471-2253-14-S1-A22

**Published:** 2014-08-18

**Authors:** Pamela Vieira Andrade, Joilson Moura Santos, José Apolinário Silva Neves Junior, José Luiz Gomes do Amaral, Helga Cristina Almeida da Silva

**Affiliations:** 1 Centre for the Study, Diagnosis and Investigation of Malignant Hyperthermia, Discipline of Anaesthesiology, Pain and Intensive Care - Surgery Department, Federal University of Sao Paulo, Sao Paulo, 04024-002, Brazil

## Background

Statins are currently the preferred agent for the treatment of hypercholesterolemia. However, myalgia, increased serum levels of creatinekinase (CK) and even rhabdomyolysis may occur, characterizing the cholesterol - lowering agents myopathy (CLAM), with a multifactorial aetiology. In statin monotherapy, the incidence of CLAM is 0.1 to 0.5 % and is dose related. RYR1 (ryanodine 1) gene alteration was observed in the patients with CLAM, an animal model of MH (malignant hyperthermia) showed hyper metabolism after simvastatin, simvastatin induced in vitro contractures in muscle of animal model and susceptibility to MH by IVCT (in vitro contracture test) was diagnosed in patients with CLAM.

On the other way, postoperative rhabdomyolysis occurred in patients treated with cholesterol-lowering drugs, leading to the suggestion of suspend these medications prior to surgery or do not use succinylcholine in patients taking these drugs . But this stance is challenged by other groups. Thus, this study examined, in rats chronically exposed to simvastatin dose previously associated with muscle alteration, the outcome of the IVCT.

## Materials and methods

With approval of the local ethics committee, 30 male Wistar rats of 8 weeks of age were divided into 3 groups: A - control, B - Statin 5 mg/kg/day; C - Statin 20 mg/kg/day. For two months , via intragastric intubations using stainless curved feeding needle, the animals in groups B and C received simvastatin daily, diluted with carboxymethyl cellulose (CMC) 0.5 %, group A received only CMC. Next, the animals were anesthetized for removal of the vastus lateralis muscles, used in the IVCT according to the protocol of EMHG. The curves for each test were analysed to determine if there were contractures.

## Results

Two animals in the control group (0.3 +0.14 g), two animals in the 5mg statin group (0.2 ±0 g) and three animals in the 20 mg statin group (0.22 ±0.02) had greater than or equal to 0.2 g contracture in the presence of 2% halothane. One animal in the 5mg statin group (0.28 g) and two group of the 20 mg statin group (0.26 +0.02 g) had greater than or equal to 0.2 g contracture in the presence of 2mM caffeine. But these contractures were minimal and there was no statistical difference in the number of animals per group with this change (chi-square, p not significant).

## Conclusions

A preliminary analysis of these findings suggests that doses of 5-20 mg/kg of simvastatin were not sufficient to cause contractures and do not increase the risk of MH crisis in healthy individuals in monotherapy.

